# Correction: Budget impact analysis of the introduction of ranibizumab's biosimilar to the Jordanian joint procurement system

**DOI:** 10.3389/fpubh.2026.1877974

**Published:** 2026-05-20

**Authors:** Jalal Abu Hamida, Nimer S. Alkhatib, Khawla Abu Hammour, Shiraz Halloush, Amanj Kurdi, Bander Balkhi, Osamah M. Alfayez, Rimal Mousa

**Affiliations:** 1 Department of Biopharmaceutics and Clinical Pharmacy, School of Pharmacy, The University of Jordan, Amman, Jordan; 2 Al-Zaytoonah University of Jordan Faculty of Pharmacy, Amman, Jordan; 3 Path Economics, LLC, Amman, Jordan; 4 Clinical Pharmacy and Therapeutics Department, Faculty of Pharmacy, Applied Science Private University, Amman, Jordan; 5 University of Strathclyde Institute of Pharmacy and Biomedical Sciences, Glasgow, United Kingdom; 6 King Saud University College of Pharmacy, Riyadh, Saudi Arabia; 7 Department of Pharmacy Practice, College of Pharmacy, Qassim University, Qassim, Saudi Arabia

**Keywords:** biosimilars, budget impact analysis, cost-effectiveness, intravitreal injections, macular edema

An incorrect **Funding** statement was provided. The correct funding statement reads:

The author(s) declared that financial support was not received for this work and/or its publication.

The original version of this article has been updated.

